# Impact of Physiological Signals Acquisition in the Emotional Support Provided in Learning Scenarios

**DOI:** 10.3390/s19204520

**Published:** 2019-10-17

**Authors:** R. Uria-Rivas, M. C. Rodriguez-Sanchez, O. C. Santos, J. Vaquero, J. G. Boticario

**Affiliations:** 1 aDeNu Research Group, Artificial Intelligence Department, Computer Science School, UNED, Calle Juan del Rosal, 16., 28040 Madrid, Spain; ocsantos@dia.uned.es (O.C.S.); jgb@dia.uned.es (J.G.B.); 2 Electronic Technology Department, Rey Juan Carlos University, c/Tulipan s/n, 28933 Mostoles, Spain; cristina.rodriguez.sanchez@urjc.es (M.C.R.-S.); joaquin.vaquero@urjc.es (J.V.)

**Keywords:** physiological sensors, affective computing, heart rate, galvanic skin response, skin temperature, emotions, applications and case studies, learning environments, feedback, open hardware

## Abstract

Physiological sensors can be used to detect changes in the emotional state of users with affective computing. This has lately been applied in the educational domain, aimed to better support learners during the learning process. For this purpose, we have developed the AICARP (Ambient Intelligence Context-aware Affective Recommender Platform) infrastructure, which detects changes in the emotional state of the user and provides personalized multisensorial support to help manage the emotional state by taking advantage of ambient intelligence features. We have developed a third version of this infrastructure, AICARP.V3, which addresses several problems detected in the data acquisition stage of the second version, (i.e., intrusion of the pulse sensor, poor resolution and low signal to noise ratio in the galvanic skin response sensor and slow response time of the temperature sensor) and extends the capabilities to integrate new actuators. This improved incorporates a new acquisition platform (shield) called PhyAS (Physiological Acquisition Shield), which reduces the number of control units to only one, and supports both gathering physiological signals with better precision and delivering multisensory feedback with more flexibility, by means of new actuators that can be added/discarded on top of just that single shield. The improvements in the quality of the acquired signals allow better recognition of the emotional states. Thereof, AICARP.V3 gives a more accurate personalized emotional support to the user, based on a rule-based approach that triggers multisensorial feedback, if necessary. This represents progress in solving an open problem: develop systems that perform as effectively as a human expert in a complex task such as the recognition of emotional states.

## 1. Introduction 

Affective computing focuses on detecting emotional reactions and then applying this information to help people properly manage their emotions [1]. This is especially relevant in the educational context, where emotions can influence engagement and performance [2]. Sensor technologies for educational context facilitate the real-time management of emotions. In fact, according to the review of the state-of-the-art done and exposed in [3], there is a gap in the research that suggests the need to improve support for sensor-based learning. A review of the state-of-the-art in affective computing in educational scenarios shows that data sources considered for affective state detection are diverse: (1) cameras for facial expressions and/or body movements, (2) pressure sensor/posture sensing chairs, (3) Kinect sensor, (4) physiological signals, such as electrodermal activity, electromyography, skin temperature, breath rate, electroencephalography, heart rate, (5) behavioural information such as keystrokes, mouse movements and pressure interaction logs, and performance features; (6) eye-tracking, (7) speech features and conversational cues, (8) text, (9) participant’s screen, and (10) learners’ answers to questions [4].

In order to obtain reliable data to improve and personalize the emotional feedback, it is necessary to explore different sensors and their use [5,6]. Since there is not a single biomechanical or physiological parameter that allows identifying the emotional state of a subject, it is necessary to use and integrate different sensors simultaneously. This approach, known as multi-sensor fusion and integration, provides more reliable and accurate information, and it has been used in many different fields [7]. Some factors affect the value of the information obtained from different sources: the validity of the signal as a natural way to identify an affective state; the reliability of the signals in real-world environments; the time resolution of the signal as it relates to the specific needs of the application; and the cost and intrusiveness for the user. These issues are to be considered in the sensor selection and system design [8]. For instance, a system implementation for determining a driver’s overall stress from physiological signals coming from low intrusive sensors tested the applicability of these methods in a real environment [9]. The results show that different stress levels could be recognized with good accuracy [10,11]. In fact, automatic stress detection is a very active research field. Related studies have shown sensors that were used to measure stress in everyday activities, which involves non-invasive sensors, optimal sensor fusion, and automatic data analysis for stress recognition and classification [12,13,14,15].

In [3], there is an extensive review of how sensor-based platforms have been used for learning support, including the affective learning domain, according to Bloom taxonomy [16]. The review presented in [3] shows that the focus on sensor-based applications for learning support is quite broad and that this support can have an effect on all the learning domains, which are cognitive, affective and psychomotor. The sensors reviewed were accelerometers, blood glucose meters, cameras, infrared cameras, electrocardiograms (ECG), electrodermal activity meters (EDA), electroencephalograms (EEG), electromyography sensors, force gauges, galvanic skin response sensors (GSR), humistors, and microphones, among others. This study reveals that most of these systems are aimed at achievements related to an active lifestyle, learning domains, formative assessment, and feedback. In addition, sensors could also be explored to recognize the emotional state of the brain in students with some mental disorder or mood disruption problems [17].

In [18], there is a survey on sensors used in a wide variety of wearable Body Area Network (BAN) systems for psychophysiological measurements. It is focused on the Autonomic Nervous System (ANS) emotional activation, emphasising how the psychophysiological responses are measured. Many works are related to biofeedback that is important to this context [19]. Various aspects, such as the choice between the special-purpose or multipurpose equipment, the obtrusiveness of the devices (wearability), potential acceptance by study subjects, connectivity, data storage, and processing capability are to be taken into account. It also highlights the design challenges to be addressed to make effective BAN systems. It concludes that the most important biosignals that can provide useful information about the cognitive and sensorial state of a person are blood pressure, heart rate, respiratory pattern and gas exchange, skin conductance, skin temperature, and electrical signals produced by the brain.

There are some works that propose additional ways and sensors for affective computing assessment in the same line of multi-sensor fusion and integration, such as manual coding of video and measuring eye movement [14,20], eye pupil diameter [20], and infrared skin temperature variations at thermally significant locations on human faces [21]. 

When focused in educational scenarios, it is necessary to define the required personalised support to be provided [22,23,24]. For this, user-centred design elicitation methodologies are needed [25]. In fact, in synthetic tutoring assistants such as those reported in [26,27], the integration of state identification and feedback could be a potential impact of robots in education related to the perception of the robot by the students to improve the learning process. Finally, there are also contributions where research has been integrated into detections of emotions to be applied at a robotic level. The projects involving humanoid robots and acquisition systems would greatly benefit from using affective learning to improve technical skills in social robotics in educational contexts. In these applications, continuous monitoring of the human psychophysiological state by the robots could be integrated to improve scenarios where humans and humanoid robots could operate [28]. It would have the chance to analyse a complete collection of data about the social environment in which the social robot is involved. According to that, this would lead to an important improvement in social robotics, making the understanding of human behaviour more reliable even in a crowded and noisy environment and in applications where long-term interactions have to be studied. 

Thus, our proposal has been focused on emotional state identification and feedback in educational contexts, considering that sensor-based platforms can become reliable learning tools capable of reducing the workload of human teachers and thus contributing to the solution of a current educational challenge. Taking into account the above, we have selected a set of physiological signals (i.e., heart rate, breath rate, galvanic skin response, and skin temperature) to *develop a sensing infrastructure that can detect changes in the emotional state of the user, and react accordingly, thus providing multisensorial feedback when needed*. This infrastructure, which is called **AICARP** (Ambient Intelligence Context-aware Affective Recommender Platform), takes advantage of environmental intelligence in terms of physiological information sources and context features in order to help the learner manage their emotional state while performing their tasks [29]. In particular, it detects changes in the physiological signals that can be associated with stressful situations, and when this happens, it alerts the learner to control their emotions and, if necessary, recommends the learner to relax by delivering modulated sensorial support. 

The first version of AICARP integrated the open e-Health Sensor Platform developed by Libelium and provided by Cooking Hacks [29]. In the second version, which is explained in detail in [30], the sensors provided by the e-Health Sensors Platform were also used. However, a new breathing sensor was added to reduce intrusiveness and improve the quality of the sensor provided by the e-Health Sensors Platform (thermocouple sensor). In addition, instead of extracting the heart rate with the ECG sensor, the eHealth pulse-oximeter was used. The second version, AICARP.V2, also allows processing information in real-time and defines a rule-based approach to trigger the multisensorial feedback to the user according to the physiological signal values that were detected. In the third version, AICARP.V3, we started using the same acquisition platform (the one from e-Health Sensors Platform using the pulse-oximeter and extended with the breathing sensor, denoted as **PeH** in this paper), but identified some issues that needed to be solved. 

To solve these problems and improve the quality of the physiological recorded signals, the **PhyAS** (PHYsiological Acquisition Shield) (previously known as AsF4eL) was designed and implemented in AICARP.V3. It has the advantage of integrating both detecting sensors and actuators into a single control unit. These improvements in the quality of the acquired signals allow for better recognition of the emotional states. The reduction on the intrusiveness diminishes the distortion that the sensors introduce in the behaviour of the user, and therefore, it reacts in a more similar way to a real stressful situation.

In this paper, we start describing the AICARP.V3 layout, breaking down its main components and functionalities. Then, we focus on comparing the usage of the two aforementioned data acquisition alternatives (i.e., PeH and PhyAS) within the AICARP.V3 infrastructure. To this end, we analyse the impact of their differences in signal quality and intrusiveness when applying the feedback delivery rule in an actual educational scenario of learning a second language. Additional benefits derived from the usage of this new version will be discussed at the end of this paper. 

## 2. Description of AICARP.V3 

AICARP.V3 has been implemented to improve the detection of changes in the emotional state of the user and accordingly provide more accurate responses through multisensorial support. Besides using PhyAS, it also improves the software tools for an expert psychologist to tag perceived emotions. This allows to support the process of detecting emotions more accurately. In this paper, we delve into this last objective of improvement. Future versions of AICARP will further progress on this improvement to provide better emotional support to the user in educational settings.

The functionality of each module in AICARP.V3 is the following:

- Acquisition: it includes the acquisition and conditioning of the physiological signals from the user by means of more appropriate sensors (pulse-oximeter, breathing belt, Galvanic Skin Response (GSR), and skin temperature) for the given task.
- Control, recording, visualization, and labelling: it contains the software to control the experiences carried out, which involves providing support for both recording the data collected and visualizing the recorded information. Thus, it implements the rule-based algorithm for automatic emotion detection and feedback delivery, and provides the tools to support the manual labelling of emotions. This task is carried out by an expert psychologist.
- Delivery: it drives the actuators that provide emotional feedback to the user. Currently, following the outcomes from the experiments carried out with AICARP.V2, in V3, we have included two emotional support devices to provide: (1) a sensorial warning (before the user gets to a critical stress level) so that the user can autonomously manage her emotional state, and (2) a relaxing feedback (when the user gets to the critical stress level) to help the user manage the emotional state. The former is a tactile actuator, which is implemented in terms of a vibrator that provides an emotional warning to the user when high physiological activation values are reached. The second consists of an array of LEDs, which is a visual actuator that supports the participant in performing modulated relaxed breathing by inhaling and exhaling following the behaviour of the provided lights, until the stress level decreases.

**Figure 1:**
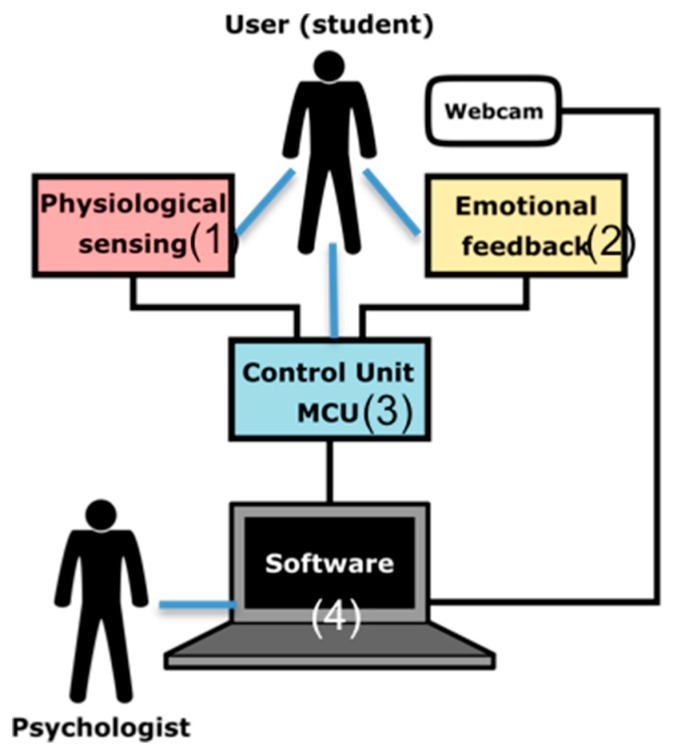
General architecture of the functional modules of Ambient Intelligence Context-aware Affective Recommender Platform (AICARP) V3.

**Figure 2:**
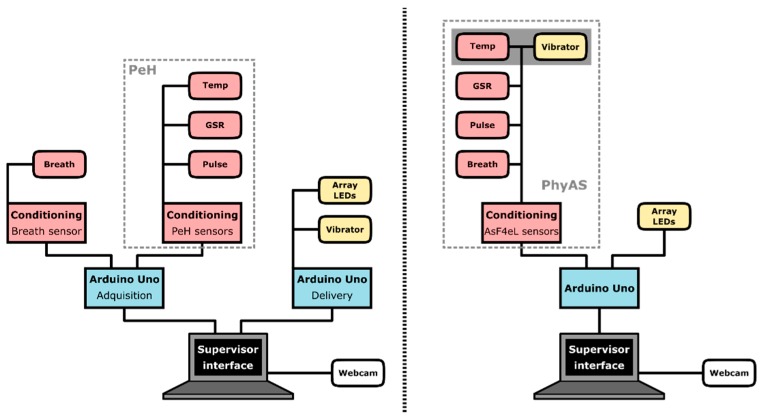
Deployment of AICARP.V3 infrastructure using PeH shield (**left**) and Physiological Acquisition Shield (PhyAS) (**right**).

Figure 1 shows the scheme of the architecture that implements the functionalities described above, which consists of: Physiological Sensing (1), Emotional Feedback (2), MCU (3), and Software Applications (4). The Physiological Sensing implements the acquisition functionalities, and it is described below. The Emotional feedback is in charge of the delivery. The software applications provide the supervisor interfaces, which perform the control, recording, visualisation, and labelling. And finally, the Control Unit acquires and processes the signals, drives the actuators and communicates with the software application. Figure 2 shows two alternative implementations of the AICARP.V3 architecture using both the PeH shield previously used in AICARP.V2 and the new shield PhyAS, which has been developed for AICARP.V3. The new shield tackles the shortcomings detected in the previous version of this infrastructure (based on PeH). One of the main differences is that PhyAS allows unifying all the sensors into a single shield, in contrast to PeH, which requires an external stage for the conditioning of the breathing signal. 

In particular:

- The capacity of the available I/O pins is greater with PhyAS since it allows connecting several actuators under a single micro control unit, MCU (the same one that acquires the physiological signals) compared to PeH, where a second MCU is needed.
- PhyAS unifies the thermometer and the vibrator in a single device. Both are encapsulated and integrated into a wristband.

Note that PhyAS incorporates a connector for the vibrator. The vibrator is one of the two actuators that has been considered in the design of the shield, as a component apart from the four physiological sensors. In addition, it is encapsulated in a wristband next to the temperature sensor. This sensor needs to be wearable and it uses few MCU resources, so it was easy to integrate it into the PhyAS. The vibrator can be replaced by another type of actuator, such as a buzzer.

Because we focus mainly on the detection part in this paper, and specifically, on how to improve the quality of the physiological signals recorded, we describe next the control unit and the physiological sensing. 

### 2.1. Control Unit (MCU)

The new PhyAS for AICARP has been designed to be used with the Arduino Uno, which is a well-known free hardware platform based on ATMEL microcontrollers. Specifically, Arduino Uno is based on the ATmega328P microcontroller, which features: 5V operating voltage, 14 Analog/Digital pins (6 PWM), 6 inputs to the 10-bit analog-to-digital converter (ADC), 16MHz clock, and communication ports, such as UART, TWI, and SPI.

As mentioned before, two microcontrollers (acquisition and feedback) were needed for the PeH, which did not allow the incorporation of new actuators. However, with PhyAS, it is possible to govern both functionalities using only one MCU since it allows for the incorporation of additional actuators. Specifically, a vibration actuator and an array of LEDs were added. The functionalities of the MCU are:

1. Communications with the supervisor interface, which controls the acquisition process and decides when the corresponding emotional support should be activated.
2. Control the recording of physiological signals at a specific sampling frequency for each signal.
3. Pre-process the physiological signals obtained from the sensors and sends this information periodically at a specific data rate to the supervisor interface.
4. Control the actuators when some kind of emotional support is delivered.
5. Control delays that may occur to maintain the flow of information in real-time.

### 2.2. Physiological Sensing

As mentioned above, this paper focuses on the comparison of two alternative modules for physiological data acquisition: PeH and PhyAS. These modules have been implemented as shields that couple into the MCU. They are designed to acquire at least four physiological signals: pulse, respiration, GSR, and temperature. 

PeH consists of a series of physiological sensors and an adapted shield. This shield contains the conditioning stage of these sensors. Only three sensors are used: pulse-oximeter, GSR, and temperature. Although PeH also incorporates a breathing sensor (thermocouple), this must be placed inside the nose, so the intrusiveness is high. For this reason, a new breathing sensor was added (piezoelectric) by means of a band on the chest. Accordingly, a shield was designed and implemented with the conditioning stage of this sensor (see Figure 2). Nevertheless, after a series of analyses of the tests carried out with PeH, several problems were identified in those three sensors, as it is described below:

- **Pulse-oximeter:** The PeH shield only linked the seven-segment display of the pulse-oximeter and used eight Arduino Uno pins (one for each segment and the eighth for sweep synchronization, as it shows more than one digit). The consequence is that we had fewer digital pins available to tackle other tasks: (i) we needed to use eight of the fourteen digital pins from Arduino Uno, and (ii) for synchronization, we had to use asynchronous pin interruption with the interruption of the implemented sampler. Then we had to add the delays of reading at a certain frequency for each digit and convert the information from seven segments to a character. Finally, it had to be converted from character string into an integer. Since the pulse sensor used is a tweezer and disables a finger, the user cannot fully use her hand to properly control the keyboard and mouse. However, AICARP should be able to be used in written educational activities, such as those reported elsewhere 2. From the above, it follows that this shield was lacking in flexibility and processing efficiency.
- **Galvanic Skin Response:** The GSR signal is amplified up to a maximum voltage of 3.3V. The MCU ADC converts analogue signals up to a maximum voltage of 5V. A large percentage of ADC levels are therefore wasted, bearing in mind that we can have a maximum of 1024 values after the ADC. This resulted in a poor signal resolution. Finally, the signal had a high noise level. These problems entail precision loss for the given problem, as it will be shown when comparing the PeH and PhyAS (see Section 3).
- **Temperature:** The temperature sensor has a long response time. For this reason, the base signal does not stabilize at the right time during the beginning of the experiment, and this affects the analysis of the data, which presents a drawback as to the precision required for the given problem.

Based on these problems, it was decided to implement an ad-hoc acquisition module, called PhyAS, which addresses above mentioned issues and provides further flexibility and functionality improvements. The acquisition signals have been designed and implemented using hardware filters. This is because the signal has a faster real-time response when it has been developed in the hardware, and the hardware processing is faster. The fact that signals can be delayed could generate a result in signal correlation with errors. All of these improvements will support a better visualization of the signal that is used to detect physiological changes, and therefore, in the process of emotional labelling, could have erroneous results. The approach consists of: (i) selecting each of the sensors separately, (ii) designing a shield for the conditioning of the breathing and GSR sensors, as well as the connection with the pulse-oximeter and the temperature sensor, and (iii) making it compatible with the MCU chosen. 

**Figure 3:**
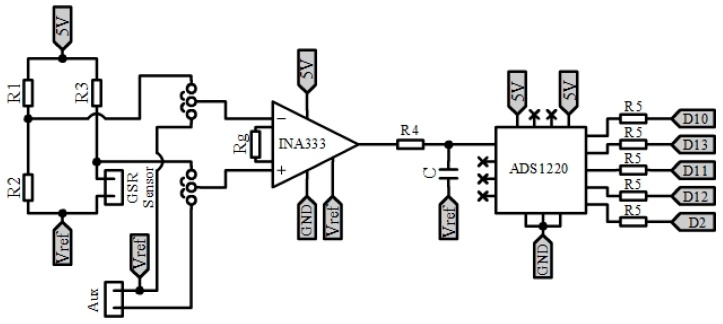
Conditioning stage of the GSR sensor of PhyAS.

**Figure 4:**
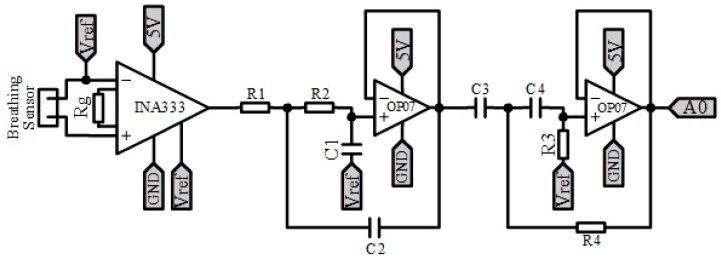
Conditioning stage of the breathing sensor of PhyAS.

Thus, the design of the PhyAS is divided into the following seven modules:

1. Reference Voltage (Vref): The PhyAS is powered from the MCU via the 3.3 and 5 V power pins. However, the conditioning stages of the breathing and GSR sensors require a reference voltage in the middle of the supply voltage. Since the MCU ADC converts analogue values up to 5 V, these stages are fed with that voltage value, so we need to generate an intermediate voltage of 2.5 V. We use a voltage regulator ISL21090.
2. Conditioning the GSR: to measure the conductivity of the participant’s skin due to sweating, we use the electrodes included in the Cooking Hacks eHealth Platform. However, we designed a new conditioning stage to improve acquisition, which is shown in Figure 3. It is powered from 5 V and makes use of the reference voltage Vref. It can be divided into four parts:
  - Wheatstone bridge: through this bridge, we convert skin resistivity values in the range of [0.12] MΩ into a signal between [−0.833, +0.833] V with respect to the reference Vref.
  - Amplification: we amplify the signal with a gain of 2.8, so we obtain a signal that varies in the range of [−2.3, +2.3] V.
  - Low pass filter: we apply a low pass passive filter at a cut-off frequency of fc = 0.5Hz.
  - 24-bit ADC converter: we digitize the signal, since the measurement range is very large for the MCU 10-bit ADC, using the 24-bit ADC ADS1220. This ADC sends the registered value to MCU through the SPI port. This element allows for acquiring the GSR signal.
3. Connection of the thermometer communication port: to determine the participant’s body temperature, we use the MLX90614 temperature sensor. This sensor does not require to be in contact with the participant’s skin, as it measures the temperature from infrared light. In addition to the body temperature, it provides support for detecting the ambient temperature. This sensor is active type FIR and has the stage of conditioning and acquisition of the signal within the package. It also processes and calculates the value of the temperatures. This sensor communicates through the I2C port (TWI) of the MCU.
4. Acquisition of the pulse-oximeter: the sensor used is from Pulsesensor. It allows being placed both on the wrist and the lobe of the participant’s ear, getting a better response with the second option. This sensor is active and encapsulates its conditioning stage, which consists of a LED diode that emits green light and, as an output, measures the amount of light reflected through a photo-receiver, which is received by an input to the ADC converter of the MCU.
5. Breathing Sensor Conditioning: The same sensor and conditioning stage that was added to the PeH is used, i.e., a piezoelectric sensor (Bionic Sleepmate). Figure 4 shows the conditioning stage. It is powered from 5 V. It can be divided into two parts:
  - Amplification: the sensor produces a voltage variation in the range of [−0.05, +0.05] V. Therefore, we apply a gain of 46 on the signal, so we get a signal that varies between [−2.3, +2.3] V with respect to Vref. To perform this amplification, we use the differential amplifier instrumentation INA333.
  - Filtering: the signal is filtered within the range of [0.1, 15] Hz through two filters, one low-pass and one high-pass, cascaded together using the Sallen-Key structure and a Butterworth approximation of order 2. The filtered signal will be received by the MCU through an analogue input. For filtering, we use two OP07 operational amplifiers.
6. Warning connection: to connect the warning device (vibrator), an MCU digital pin has been assigned to perform the Trigger function.
7. Connectors: Jack connectors are added for both the vibrator and all sensors except for the GSR, which incorporates a special connector.

To implement the printed circuit board (PCB), the following phases were carried out:

(i) A circuit was implemented with each one of the modules
(ii) The correct functioning of the circuit was verified
(iii) The layout was designed with the KiCad tool
(iv) The shield was routed with the LPKF Protomat C60 CNC milling machine
(v) Each one of the components was soldered to the PCB

**Figure 5:**
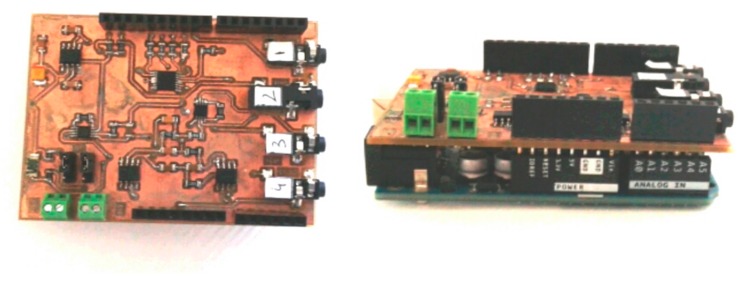
PCB of PhyAS for physiological signal acquisition, compatible with the Arduino Uno MCU.

Figure 5 shows the final implementation of the PhyAS shield.

## 3. Results

Our research question here is *how to improve the quality of the recorded signals for better emotional detection so we can implement a more accurate rule-based trigger that supports more personalized feedback to the user while dealing with stress in an educational setting where ambient intelligent features are appropriate*. Thus, first, we needed to check if the new acquisition shield (i.e., PhyAS) obtained physiological signals with better quality than the previous PeH. Then, we applied the feedback delivery rule to data collected with both deployments and analysed the impact on delivering emotional support. To perform these analyses, we needed to evaluate both modules with the same time-frame approach. Notice that, as in [29,30], the educational tasks were oral, as the PeH includes a pulse-oximeter from where users cannot be asked to type. 

### 3.1. Experimental Setup and Execution

This experiment took place in an educational scenario of learning a second language. It consisted of three oral tasks in a non-native language for the participant, in our case English. It was chosen to recreate the setting and experience lived by the user when they are examined in a non-native language in the learning context. The tasks that were to be carried out consisted of: (i) Reading a plain text (Task 1), (ii) Exposing a specific topic orally (Task 2), and (iii) Exposing a specific topic orally but with the restriction of using twelve words, which they have to memorize, because they are only present before the exposition time (Task 3). In addition, before the first task, and following the best practices in physiological data acquisition [31], the user was asked to relax for two minutes to measure the user initial baseline. Similarly, at the end of the three tasks, the user was asked to relax again to measure the final baseline. 

**Figure 6:**
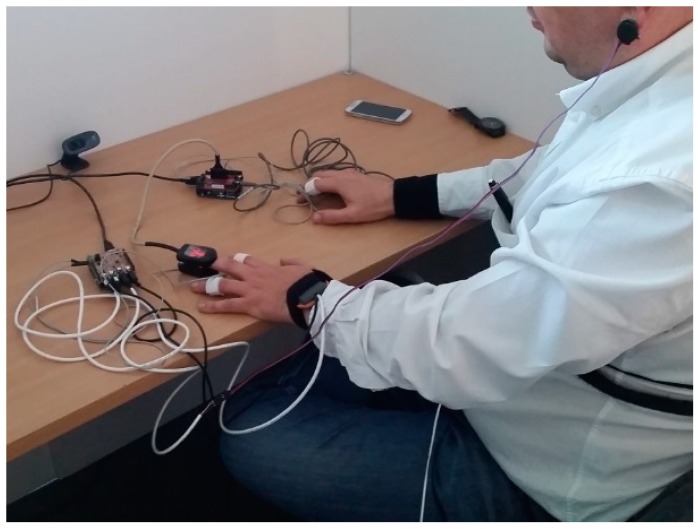
Participant with the AICARP.V3 with PeH and AICARP.V3 with PhyAS.

Table 1 shows the different number of experiences that we have carried out since we started designing and developing AICARP.V3. A few tests failed due to conditions outside the system as the sensors were not connected correctly. At the end of these experiences, the system was undergoing improvements until we developed the current system which is presented here. Then, we compared both platforms at the same time in the experiment reported next. The participant was a 38-year-old male. He wore both systems (i.e., AICARP.V3 with PeH and AICARP.V3 with PhyAS) on his body, as shown in Figure 6. There are other works [32,33] where a single subject has also been considered to validate the functioning of the system in order to contrast its validity. Reliability of the experiment on a single subject can be ensured by using reliable instrumentation, repeated measurements, and also by describing the experimental conditions in detail (i.e., the measurement conditions and the nature of the treatment) [34]. We considered this kind of validation, and the variability of the subjects did not influence the outcome of the comparison at hand. What has been tried is to demonstrate the improvement in the functioning of the hardware and software solution developed (PhyAS) compared to a previous approach (PeH).

During the experiment carried out to compare both PeH and PhyAS, the participant’s heart rate, galvanic skin response, skin temperature and breath rate were simultaneously recorded using both deployments. First, the participant was asked to relax for two minutes before starting the experience. This aims at obtaining the current baseline of the participant that models his physiological state, and this is useful to deliver the feedback in terms of a trigger-based rule, as commented below. After the baseline, the participant was asked to perform the three tasks as follows. In Task 1, the participant receives the instructions of the task, which consists of reading a text twice. The first reading is done in silence to understand the statement. The second reading must be done aloud since the ultimate objective is to support the training of non-native speakers. In Tasks 2 and 3, the participant receives the instructions of the task, which consists of talking aloud for five minutes about a specific topic. He had one minute to think what to say. After that, the participant was asked to relax again for another two minutes. These tasks were made using reliable instrumentation, repeated measurements, and also by describing the experimental conditions in detail during the evaluation process of the hardware (approximately seven days).

### 3.2. Quality of Signals Collected by PeH and PhyAS

Table 2 shows the different components used in both the previous and new version of AICARP acquisition shields, which both have the same functionality. 

Next, we analysed each of the sensors that were changed from PeH to PhyAS: (i) GSR sensor, (ii) pulse-oximeter, and (iii) temperature sensor. 

#### 3.2.1. GSR

To analyse the GSR sensor, we studied analytically the exploitation of the ADC, as well as the resolution of the signal. Subsequently, the noise level on the simultaneously recorded signals was studied. For this, we followed the next steps:

1. Analytical analysis of the stage of conditioning in a steady state, which allowed us to study both the use of the ADC and the measurable range for each acquisition module from its formulation.
2. We studied the resolution of each acquisition module in the measurable range of the signal.
3. We characterized the noise level present in PeH.

Table 3 summarizes a comparison of the conditioning and acquisition of GSR signals for both PeH and PhyAS. On the one hand, it should be noted that while the PeH conditioning stage linearizes the skin conductivity (CGSR), in PhyAS the resistivity (RGSR) is linearized to a greater degree. It was decided to approximate it to a straight line since it does not have a significant impact on the signal resolution. On the other hand, conductivity is the inverse of resistivity. Therefore, it is easy to get the value of one from the other. However, it must be kept in mind that the inverse value responds to an equilateral hyperbola function, where there is no linearity. 

Table 4 shows the formulation that allows understanding the behaviour of these schematics. In the development of the schematic, we have done an analytical analysis in a stationary state.

**Figure 7:**
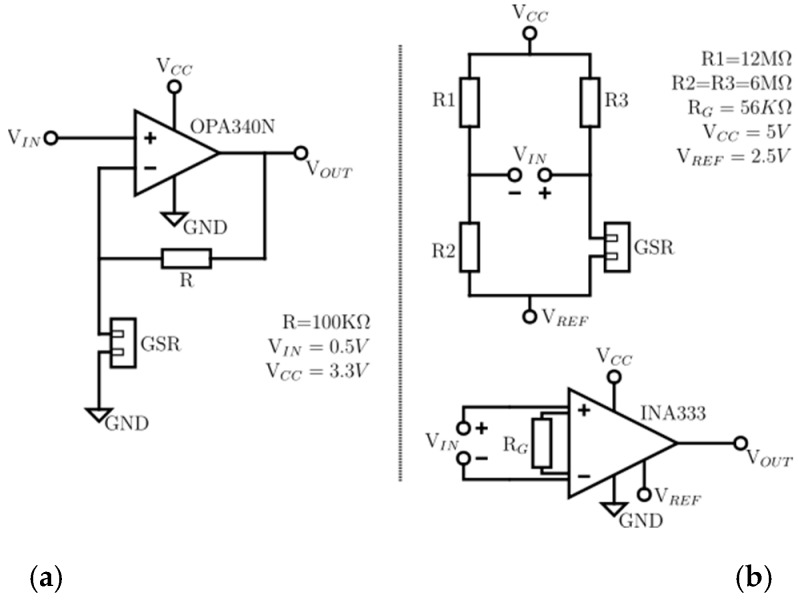
Conditioning stage, steady state of the GSR signals for both acquisition modules: (**a**) PeH, (**b**) PhyAS.

In Figure 7, the GSR schematics are simplified to a stationary state, for both PeH and PhyAS. Table 3 shows the formulation that allows us to understand the behaviour of these schematics.

**Figure 8:**
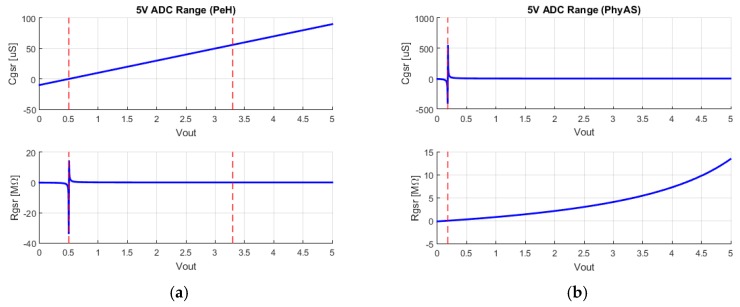
Acquisition of GSR signals for both acquisition modules: (**a**) PeH, (**b**) PhyAS.

Figure 8 shows the possible skin resistivity and conductivity values in the ADC conversion range. This is used to identify the measurable values for each of the GSR sensors. 

First, we can observe the offset of the signals. This offset is estimated as a function of the voltage value from which negative conductivity values are obtained. In addition, in the case of PeH, the voltage of 3.3 V is identified, which is the saturation value of the signal in the amplification stage. Therefore, we can determine the measurable range of each signal for both modules and calculate the percentage of ADC utilization. If we take into account the level of quantification of each of the ADC converters together with the use of the ADC, we can obtain the amount of values that we can measure within the measurable range for each of the signals. All of these results are summarized in Table 5.

This information can be summarized in the following conclusions:

- The PhyAS conditioning stage is best suited to the signal acquisition stage. This is because both stages work the signals in the same voltage range. For this reason, PeH wastes 34% of the ADC quantification levels. In addition, both platforms add an offset to the signal that also wastes part of the converter, with PeH being the most affected by 10% and PhyAS by 3.58%. This means that the percentage of the ADC quantifier levels used for PhyAS is 96.42%, whereas for PeH it is 55.96%.
- The number of measurable values is 28,234 times greater for PhyAS than for PeH due to the 24 bits conversion of PhyAS versus 10 of PeH.
- The range measurable by PhyAS comprises eight orders of magnitude, whereas PeH comprises three orders of magnitude.

The number of measurable values after the acquisition stage over the measurable range of the signal has a direct impact on the acquired signal resolution. In order to study the resolution of the measurements acquired after the ADC of each of the modules, we used the following procedure: we obtain the possible quantified Vout values and calculate the possible skin resistivity and conductivity values. In this way, we can calculate the resolution of the acquired signal, ∆RGSR and ∆CGSR, where the only totally linear output is that of the CGSR of the PeH module and the signal with the highest linearity for the PhyAS module is RGSR. Table 6 shows the maximum, minimum, mean, and standard deviation values of these signals for both acquisition modules.

From this information, it can be concluded that the main differences between PhyAS and PeH regarding GSR are the following:

- The skin resistivity measurement of PhyAS can be considered linear, since the standard deviation is 0.6 Ω, i.e., a value very close to 0.
- In order to compare their resolutions, their mean resolution value will be taken into account since we are comparing linear responses with equilateral hyperbolic functions. If we focus on resistivity resolution, we can see how PhyAS is five orders of magnitude smaller than PeH. However, focusing on the resolution of conductivity PeH is two orders of magnitude smaller than PhyAS.

**Figure 9:**
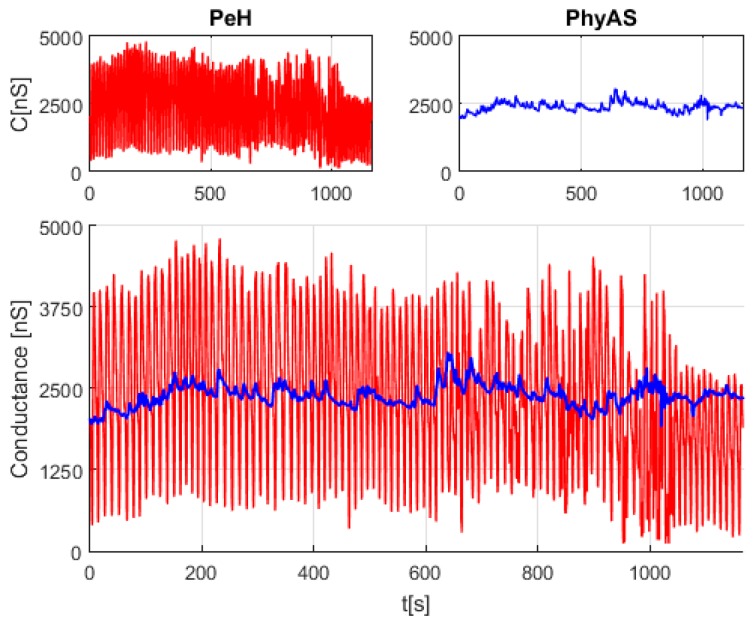
Conductivity of the skin acquired simultaneously with both acquisition modules.

Finally, Figure 9 shows the signals acquired with both acquisition modules simultaneously. It can be seen that the signal coming from the PeH module contains a high level of noise, whereas the signal coming from PhyAS provides smaller variations in the signal, which may contain useful information in the detection of emotions. 

**Figure 10:**
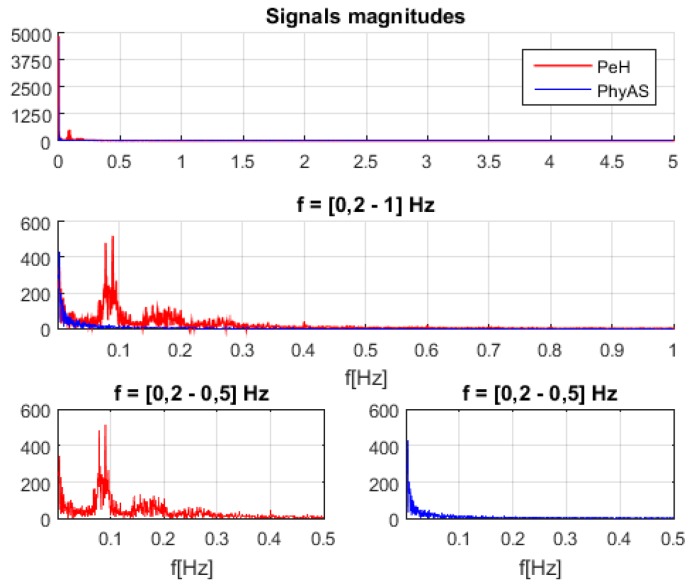
FFT of the conductivity signals for the PeH and PhyAS modules.

Notice that both stages of conditioning have a low pass filter of the first order. In the case of PeH, it is active with a cut-off frequency of approximately 16 Hz, whereas in PhyAS, it is passive with a cut-off frequency of approximately 0.5 Hz. However, the noise frequency components present in the PeH fall within the pass band, as shown in Figure 10. Specifically, at frequencies of 0.0718 Hz and 0.0819 Hz. These components are not found in the PhyAS signal, where we only have the continuous component of the signal.

We then focus on characterizing the noise level present in PeH. Table 7 shows the maximum and minimum amplitude, as well as the mean amplitude and standard deviation for the PeH CGSR signal. The number of levels and percentage of the ADC converter of the noise over the measurable range of the signal are also shown. We can point out that the noise level present in the PeH signal varies in the 20-40 level range of the ADC, which affects the information collected by the sensor.

#### 3.2.2. Pulse-oximeter

The objective of this evaluation is to demonstrate that with the new pulse-oximeter sensor, which solves the intrusion problem present in the PeH sensor (i.e., disables the finger), there is no signal quality loss. In order to face their main difference, which is that the PeH sensor provides the average value over the pulse changes whereas PhyAS gives the instantaneous value, we average the PhyAS signal in a range of window sizes, from which we are able to study the correlation between the PhyAS and PeH signals and the delay caused by the averaging. All this allows us to determine the best window size to average the pulse changes obtained by PhyAS.

Table 8 summarizes the main characteristics of the pulse signal for both acquisition modules. Here, the PeH average pulse values (BPM) are considered along with the PhyAS instantaneous values. This allows the latter to approach the R-R interval of the ECG. However, the instantaneous data are expressed in bpm. 

**Figure 11:**
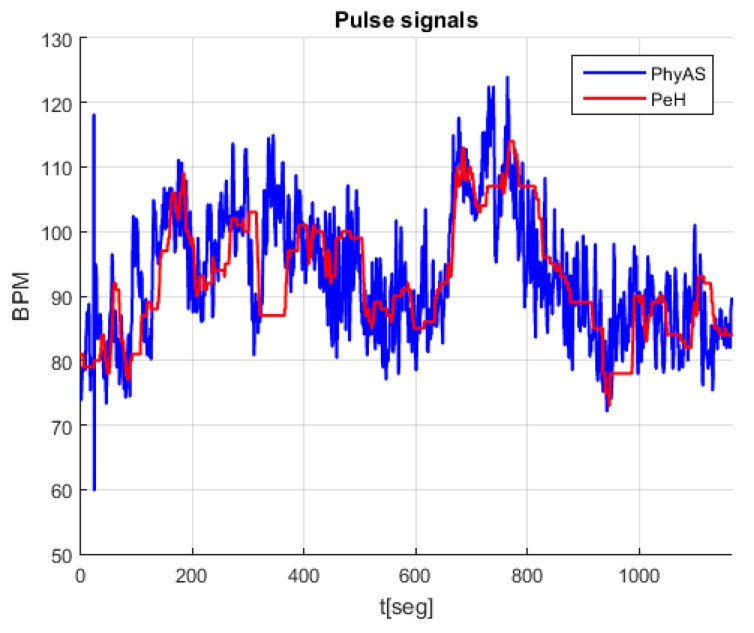
Pulse signals recorded during the experiment.

Figure 11 shows the signals acquired simultaneously with both modules, where we see that the pulse signal of PhyAS oscillates more frequently as it provides instantaneous values. However, on average, it seems to behave like the pulse signal of PeH.

According to these results, we want to average the PhyAS pulse signal so that it resembles PeH as closely as possible. However, this averaging will be done with a window whose size is determined by the number of changes in pulse. So, the time size it covers will depend on that mean value, where the higher the mean rate value, the smaller the time window size and vice versa. This is important because the only real-time averaging is the one that depends on past elements. This causes a delay in the signal that will depend on the size of the time window, which is dynamic. 

**Figure 12:**
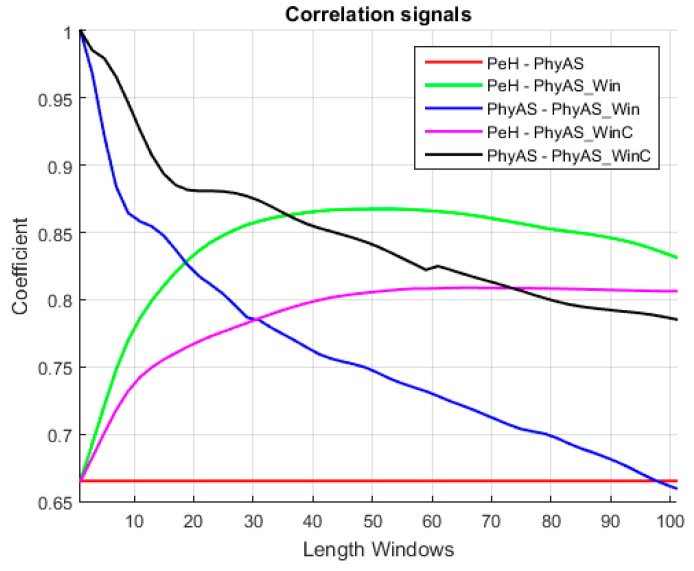
Correlation between the original signal of PhyAS, the different window sizes for the averaged and, the signal to compare PeH.

The resulting signal has been calculated in the range of 1 to 101 pulse changes. From this, we can determine the window size that provides the highest correlation with PeH behaviour. This resulting signal is called PhyAS_Win. In addition, we consider a centred window called PhyAS_WinC in order to check if the PeH signal is delayed in time because this signal should not be delayed with respect to PhyAS. Figure 12 shows the correlation of each window length calculated with the original PhyAS and the PeH signals. In addition, the initial correlation of the PeH and PhyAS signals is shown as a constant.

The initial correlation between the PeH and PhyAS signals has a value of 0.67. If we look at the correlations with the averaged signal, we see that the PeH signal is averaged since the smoothing obtained with PhyAS_Win and PhyAS_WinC tends to look more like PeH than PhyAS. Moreover, there is a delay in the PeH. If we focus on the correlation of PeH with PhyAS_WinC, we see that it tends to have less resemblance than when correlated with PhyAS_Win. This delay is also seen in the PhyAS correlations, where the correlation of PhyAS with PhyAS_WinC tends to be more similar than when correlated with PhyAS_Win. This is caused by the signal delay, as both averaged signals are smoothed to the same degree.

Finally, the correlation between PeH and PhyAS_Win grows more pronounced to a window of about 20 in size. From this value, it grows more smoothly to reach the maximum value for a window of about 51 in size. In addition, PhyAS_Win begins to look more like PeH than PhyAS for a window size of about 19.

**Figure 13:**
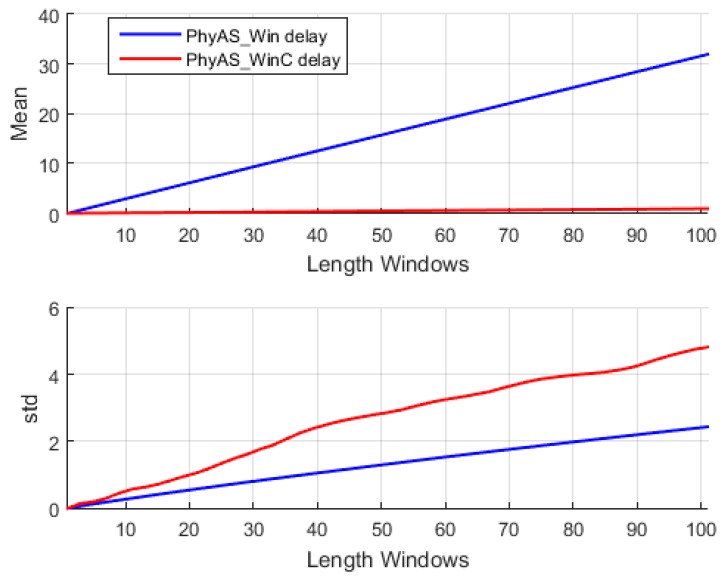
Statistics of the signal averaged, PhyAS_Win and PhyAS_WinC.

Figure 13 shows the mean delay of the averaged signal as a function of window size, as well as the standard deviation of this delay for the PhyAS_Win and PhyAS_WinC signals. The mean delay of the signal averaged with a centred window is not zero, although it tends to be. This is because the time window of past events does not have to be the same as those of future elements. Therefore, the signal will have small overtures and delays over the flow of information.

**Figure 14:**
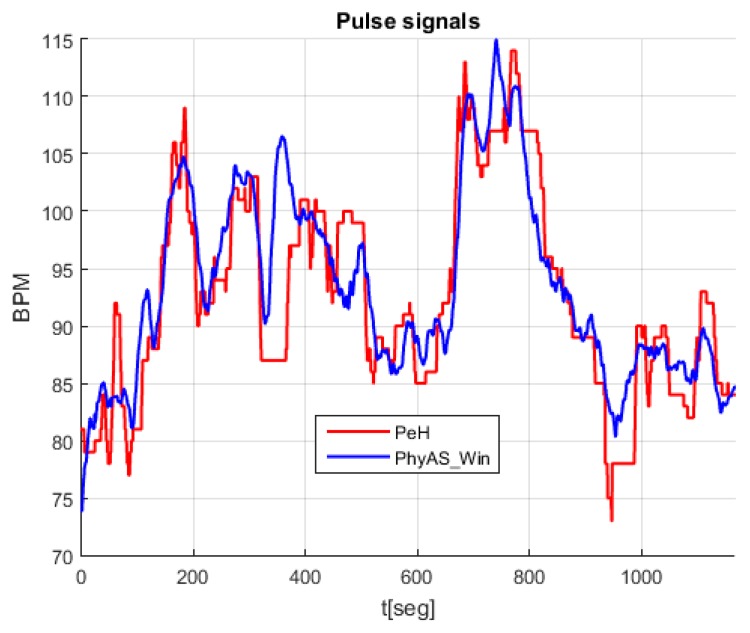
Comparison between the averaged signal, PhyAS_Win, with a window size equal to 21 pulse changes and the PeH signal.

It can be seen (Figure 14) that the average delay and the range in which it varies becomes greater as the window size increases. Accordingly, we can consider a window size of 21 to average the signal of PhyAS, since it is this value from where the correlation with PeH increases sharply. This way, there will not be as much delay as with a 51-window size, which corresponds to the maximum correlation. It is also noticeable that the correlation of the signal averaged with PhyAS and PeH is practically identical, i.e., it resembles both the PeH signal and the PhyAS signal. In Figure 14, we can see the comparison between the averaged signal with a window of size 21 and the PeH signal.

Therefore, with the incorporation of the new sensor, we are not losing quality in the signal; in addition, we have a higher resolution. It should also be noted that by obtaining the instantaneous value, we can adapt the window size to our convenience, which in essence provides us more flexibility to deal with the particularities of a given situation.

#### 3.2.3. Temperature

The objective of this evaluation is to characterize the response time of the temperature sensor, which is obtained from a new experiment that allows us to measure the signal when the sensor is placed and removed, thus being able to check the behaviour of the sensor when there are sudden changes in the measured temperature. In addition, the body temperature value was periodically collected from a pharmacy thermometer during the experiment. The steps that were considered are as follows:

1. The offset present in the signals is corrected in order to determine the response speed and correlation with the pharmacy thermometer.
2. The noise level is characterized.

The ambient temperature of the PhyAS sensor will not be taken into account in this study since PeH does not have this measurement. Table 9 summarizes the main characteristics of the conditioning and acquisition of temperature signals for both PeH and PhyAS. Notice that the conditioning and acquisition stage of the PhyAS temperature sensor is inside the sensor. This sensor returns the temperature (of the object and of the ambient temperature) as requested by the I2C communication port. 

**Figure 15:**
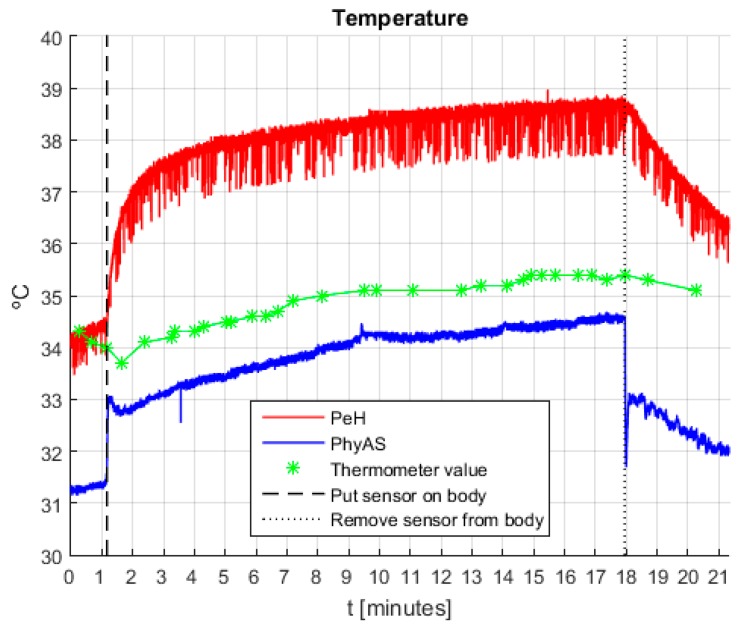
Temperature signals recorded during the experiment.

Figure 15 shows the signals obtained during the experiment, where it is necessary to consider: (i) the asymptotes represent the instants in which the sensor is placed or removed from the body, and (ii) the values recorded by the pharmacy thermometer are marked with the star sign (‘*’) and are linearly joined. 

**Figure 16:**
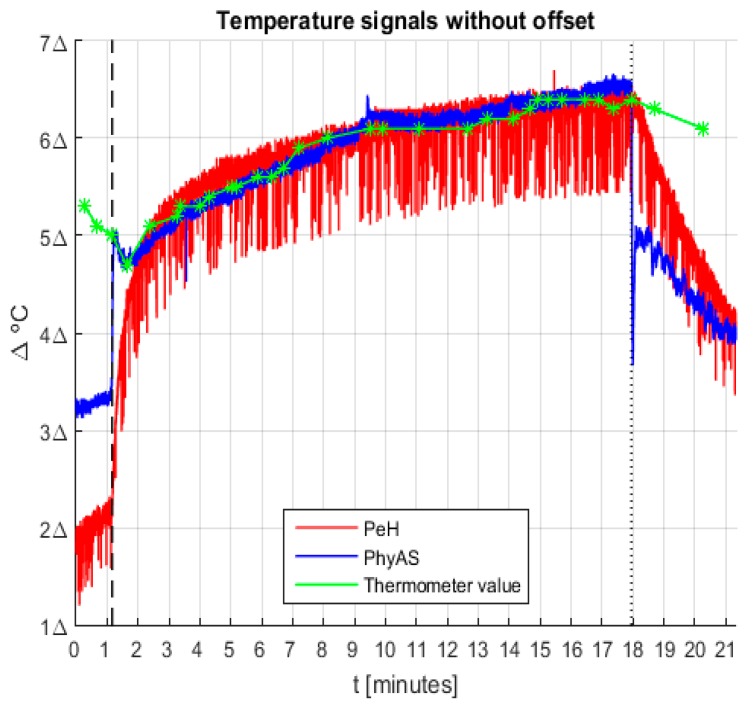
Temperature signals recorded during the experiment without offset.

First, we correct the offset of the signals because, if we compare their value with that given by the pharmacy thermometer, it can be seen that the average value of PeH is roughly 3.2 °C higher, whereas with PhyAS is around 0.9 °C inferior. In Figure 16, the signals acquired with the offset correction are shown. It shows that the signals behave very similarly over the time the user is wearing the sensor. However, we can see how the response time of PeH is slow compared to PhyAS. We should also point out that the signal increase that occurs after placing the sensor is less for PhyAS than for PeH. These behaviours are summarized in Table 10. 

From these data, we can draw the following conclusions:

1. Both signals are similar to the pharmacy thermometer signal. However, the correlation of PhyAS is higher and its value is increased in roughly one unit (0.986) with respect to the correlation value of PeH (0.805).
2. The response time of the sensor is improved. While in PeH, it is needed to wait a little more than a minute until the sensor value stabilizes at body temperature, usingPhyAS the waiting time is significantly reduced to a little more than three seconds.

Finally, we see how the noise level present in PeH is higher than that of PhyAS. Table 11 shows the mean value and standard deviation of the noise level present in the signals. From these data, we can conclude that on average, the noise level of PeH is three times higher than that of PhyAS. This value is half in the best case but more than eight times higher in the worst case.

### 3.3. Comparison of the Feedback Delivery Rule with PeH and PhyAS

Once we have confirmed that the PhyAS provides improvements in the quality of GSR, pulse, and temperature signals, we would like to evaluate if this has a positive impact when generating the delivery of the emotional support. 

AICARP.V3 follows the same feedback approach as AICARP.V2, which is based on using a rule-based trigger that drives when the different types of feedback are provided, as is described in [30]. This is a parametrized rule which consists of the combination of the physiological signal values in order to define the activation level of the user, so that first the warning is provided and, when needed, there is a recommended breathing support (i.e., relaxation feedback). 

The rule has two sets of parameters that must be adjusted through modelling, which is currently done with the involvement of experts, but in the future, our objective is to compute the best trigger values for a given user in terms of machine learning techniques following a similar approach to the one that was implemented in [35]. In each set there is a parameter for each sensor, which we will call S_i where i is the integer that the sensor represents:

- The first set of parameters is called *%i*, and it indicates the percentage of distance where the maximum value is with respect to the sensor average baseline value. Thus, the maximum value obtained for providing feedback to the participant is this percentage, which is based on the signal value. This percentage is applied to the average of the signal along the initial baseline.
- The second set of parameters it is called *α_i_*, and it is the weight factor said that is applied to each signal sensor. This way we are able to control the relative importance of a specific signal for a given participant, which allows us to personalize the behaviour of the function that determines when to deliver a warning or relaxation feedback for a given context and user. The sum of the weight factors of the four sensors must be 1:
  $$\sum_{i\;=\;1}^{4}\propto_{i}=1.$$

The rule that drives the delivery behaviour is based on the values obtained from the physiological sensors, which are collected over the baseline phase, i.e., pre-test, which precedes the test task. Here, the average of all these values is taken as the reference of where to give the recommendation to the participant. The rule is defined in the following steps:

1. During the Baseline phases, the values read by each of the physiological sensors are gathered.
2. After finishing the baseline, two parameters are calculated for each of the sensors: (i) $m_{i}$, the average of each sensor values that were captured during the baseline, and (ii) ***max_i_***, the maximum value that each sensor has reached above the baseline average, and is given by the following equation:
  $$max_{i}=m_{i}*\frac{{\%}_{i}}{100}.$$
3. During the Task performing phases, the value of the function $f_{k}$ that determines whether or not to give a recommendation is calculated, where *k* corresponds to the given moment. The final value of *f* depends on the normalized distance with respect to the mean, $d_{i,k}$, which has the value of the signal at the given moment, denoted as *S_i,k_*. The following equations describe the approach:
  $$d_{i,k}=\frac{S_{i,k}-m_{i}}{max_{i}}\;f_{k}=\sum_{i\;=\;1}^{4}d_{i,k}*\propto_{i}.$$

The recommendation warning is activated if the value of $f_{k}$ is greater than 0.7 and less than 1.0. The value of 1 is considered because the values are normalized as a function of the maximum distances. The value of 0.7 was set because the warning recommendation should be provided from a lower than the maximum, i.e., 1.0, when the mitigation recommendation is given. The value of 0.6 creates a margin with respect to the value 0.7, thus providing a certain hysteresis. Once the warning is activated, the possibility of reactivating is blocked until the value of $f_{k}$ decreases to values lower than 0.6, where it is unblocked, and a warning is given again if it exceeds the value of 0.7 again. 

The relaxation feedback recommendation is activated if the value of $f_{k}$ exceeds the threshold of 1.0 at a given moment, and it will be maintained until $f_{k}$ is less than 0.7.

The reason for defining an activation threshold and another threshold for deactivation is to create a margin, or hysteresis. This margin would prevent the recommendation from changing its state continuously when values oscillate around the threshold value.

The warning signal lasts a brief time instant, just a few seconds, in contrast to the relaxation feedback, which remains active for as long as $f_{k}$ meets the condition which corresponds to having the relaxation feedback activated.

**Figure 17:**
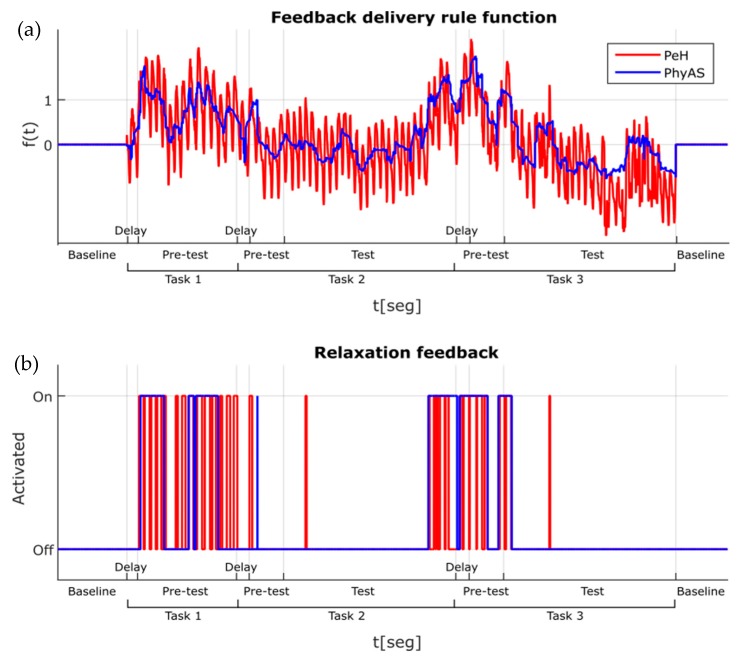
Feedback delivery rule function: (**a**) Feedback delivery rule function, (**b**) Relaxation feedback.

The configuration parameters were as follows: the percentages chosen raised 20% from the baseline average, thus following what was done in [27], and the weights correspond to an increase of 25% to give the same importance to all the sensors. Although it is expected that both functions look the same, since they are measuring the same physiological reactions, it can clearly be seen that the one from PhyAS is more stable. In particular, the noise present in some sensors (GSR to a greater degree and Temperature to a lesser degree) affects the function *f* (see Figure 17). While in PeH, the function oscillates with great amplitude in PhyAS and we have a softer signal. In Figure 17, where the aforementioned tasks are depicted, it can be noted that delay corresponds to the time needed to give the instructions to the participant. Here, the pre-test corresponds to when the user reads the instructions and test to when the user performs the tasks (only for tasks 2 and 3, since task 1 is only reading aloud the given text for calibration purposes). 

The impact of the above is noticeable in the second graph (relaxation feedback) of Figure 17, where avoiding some isolated feedback of short duration:

1. PeH only provides small pulses very often, which is why we had to add a time delay when vibration feedback was given.
2. PhyAS, however, provides longer duration pulses which encompass (or collect) the small concentrations of PeH pulses.

Thus, it can be concluded that the improvements in the physiological acquisition has a positive impact on the emotional support to be provided since the emotional feedback can be delivered at the appropriate moments. 

## 4. Discussion

Affective domain in education refers to the approach in which students deal emotionally with values, feelings, motivations, and attitudes, which affects their performance [36]. This mastery is often categorized according to the complexity of the behaviour incorporated by the subject. In this context, we have developed the AICARP platform with custom hardware and software to improve support for sensor-based learning. In this proposal, a platform based on sensors can become a reliable learning tool to contribute to the solution of the educational challenge of integrating the detection of emotions in learning. 

However, during the experiments carried out, we identified the following drawbacks in the acquisition stage, which lead us to work on further improvements that have been resolved:

(i) Intrusiveness caused by the pulse sensor, which was based on a clamp that prevents the user from using their finger. This presents a problem while performing common educational tasks, where the user needs to type into the computer because writing is required.
(ii) Poor resolution and low signal-to-noise ratio (SNR) in the acquisition of the GSR signal.
(iii) Slow response time of the temperature sensor.

The key issue of this paper is that ***the improvements done in the third version of AICARP to its detection capabilities means that the system response will better resemble the human response in a complex task such as the emotional labelling***, which involves detecting, labelling, and real-time identification of the moment in which the emotional state of the user changes. According to results, AICARP.V3 is less intrusive, more easily portable and “connectable” to the user. In particular, in this paper, we have improved the detection shield, as described in Section 3. Thus, we have substituted the PeH shield by the PhyAS shield. The validation of the usage of both PeH and PhyAS within AICARP.V3 is summarized in Table 12:

The new platform aims to solve some problems found in the pulse, GSR, and temperature sensors of the PeH platform. The pulse sensor is changed by an ear clip sensor, which allows the fingers to be used in new possible tasks, such as written tests, and in this way, to decrease the intrusiveness. For the skin conductance sensor, the conditioning and acquisition phase is developed in order to collect the signal with greater precision. The temperature sensor is changed by another one with a low response time, and also, measure the temperature both in the skin and in the environment, so we can also deal with the effect of the environment on the skin temperature. PhyAS could give instantaneous changes with a similar behaviour to the HRVs (Heart Rate Variability) from an electrocardiogram. For the GSR, PhyAS provides results with more precision and fewer errors. In the case of temperature, PhyAS has a better response and less noise. In order to improve the analysis of the GSR version, the tools and algorithms described in [14,37].

As it is described below, the results from this analysis lead us to conclude that AICARP.V3, based on PhyAS, has a positive impact on the emotional support to be provided, because this feedback can be delivered at the appropriate moments. All of these improvements support a better visualisation of the signal that is used to detect physiological changes. Moreover, these changes provide an advantage point in using AICARP.V3 in detecting physical and emotional states that impact on performing while involved in learning environments. The improvement of the new version of AICARP provides a more accurate system´s response, which addresses a critical open issue, i.e., to have a shield that performs as effectively as the human expert in a complex task such as emotional detection. Furthermore, the system provides support for detecting, labelling and responding in real-time to the moment when the emotional state of the user changes. In fact, it could be applied for new environments and experiences in the context of learning. For instance, writing exams, driving exams, job’s interviews, presentation in conference/seminars, etc.

From the educational perspective, it can be discussed that for the particular participant who took part in the experiment described here, feedback seemed to be more relevant when the user was preparing the tasks to be done, instead of when actually doing them. Further experiments involving psycho-educators are required to gain new insights into the educational rationale. This is out of the scope of the electronics of the system, which is the key issue evaluated in this paper. 

From a broader perspective of the AICARP functionalities in the educational domain, it could be discussed that there are critical problems to be addressed, building on this study’s outcomes. First, so far, the system provides two types of feedback according to [3]: “How am I going?” and “Where-to next?”. The former is treated here through the warning signal with a meta-cognitive consequence, meaning that the purpose is to make the user aware of their own level of stress so that performance might be affected. The latter is more focused on mitigation behaviour, which in this case is implemented through the relaxing feedback when the stress level is already too high. The advantage of the current version is that it is able to better detect when the user is getting stressed, which could also be used in predicting performance in self-regulated learning [36]. As to the educators’ viewpoint on this type of functionalities, there is a gap to be filled in terms of helping them understand the possibilities coming from the new affordances [38]. 

Finally, there is the ultimate system objective from the detectors’ standpoint, i.e., human experts who label affective reactions, which is to resemble as much as possible their detection behaviour. It is arguable that with a continuous application of the system to a given learner, AICARP might outperform those experts. To this, our next step is to improve the rule that triggers the feedback so that the weights that control the value of the different signals in detecting a particular user stress would vary over time taking into account the evolving nature of each person’s behaviour. To this end the rule that controls the feedback must be adjusted through modelling, so the best trigger values for a given user can be obtained from machine learning techniques following a similar approach to the one that was implemented in [35]. 

## 5. Conclusions 

Physiological data such as pulse, breathe rate, skin temperature, and skin conductance can provide valuable information to identify changes in the user emotional state. Thus, sensors are to be as accurate as possible to collect this information. We have proposed the *AICARP platform to support the acquisition of physiological signals and use that information to deliver personalized multisensorial emotional support to the user*. 

In this paper, we present the *improvements we have made in the acquisition shield to improve the quality of this information*. In particular, the new shield, called PhyAS, reduces the number of control units while gathering the physiological signals with better precision and with less intrusiveness. It also allows more flexibility in the incorporation of new actuators. Thus, we have presented AICARP.V3 and compared the new acquisition shield (PhyAS) with the previous one (PeH) that consisted of open hardware distributed by Cooking Hacks. PhyAS improves the precision of the GSR signal, reduces the intrusion of the pulse sensor and improves the response time of the temperature sensor. We could use a low pass digital filter because the calculated signals are processed by software. However, this could generate a delay in the signal and extra computational time. On the other hand, this improvement is not only intended to solve problems in the signal, but also the intrusiveness and response time. Intrusiveness caused by the pulse sensor, which was based on a clamp that prevents the user from using their finger. This presents a problem while performing common educational tasks, where the user needs to type into the computer because writing is required. Using both shields simultaneously with a user performing some emotionally challenging learning activities tested these improvements. 

We have also analysed the impact of their differences on signal quality when applying the feedback delivery rule in a real educational scenario of learning a second language. In this particular case, for a given user, feedback seemed more relevant when the user is preparing the tasks to be done, instead of when actually doing them. To get a deeper insight into this issue, from where to improve performance, we are planning to carry out another study with some psychologists, and design an experimental setting with their support that takes into account relevant methodological variables, as we have done in the past (see [2]). Moreover, using data mining with the most continuous and lasting tracking of a single individual could lead to finding out the most appropriate value for a given person.

## Figures and Tables

**Table 1:** Number of experiences with both platforms.

	Successful Tests	Labelling Post-Test	Age	Sex	Number of Participants
2015–2016	14	12	14–19	Male (9) and Female (5)	14
2016–2017	6	5	20–35	Male (3) and Female (3)	6
2017–2018	10	10	12–17	Male (4) and Female (6)	10

**Table 2:** The different tested modules for the components of AICARP.V3.

Component	PeH	PhyAS
MCU	ATmega328	ATmega328
Shield	Based on Libellium board	Customized design
Pulse	Pin pulse-oximeter by Cooking hacks	Ear-ring pulse by Pulsesensor
GSR	Electrodes and conditioning shield by Cooking hacks	Electrodes by Cooking hacks and customized conditioning shield by
Temperature	RTD by Cooking hacks	MLX90614-FIR
Breathing	Bionic + PCB with customized design	Same as PeH

**Table 3:** Comparison of the different GSR designs.

Step	PeH	PhyAS
Sensor	Same electrodes to acquire signal	Same electrodes to acquire signal
Signal conditioning	The stages are a voltage reference for the signal offset, an operational amplifier (low pass filtering and amplification).	The stages are: a Wheatstone bridge to measure the resistivity of the skin, a gain stage using an instrumentation amplifier (INA333) and a first order passive low pass filter.
Linearisation	Conductivity of the skin	Resistivity of the skin
Acquisition	Arduino ADC (10 bits, 5V)	ADS1220 ADC (24 bits, 5V)

**Table 4:** Formulation of the different designs.

PeH	PhyAS
$V_{IN}=0.5\;V$	$V_{IN}=\left(V_{CC}-V_{REF}\right)\cdot \left(\frac{R_{GSR}}{R3+R_{GSR}}-\frac{R2}{R1+R2}\right)$
$V_{OUT}=C_{GSR}\cdot V_{IN}\cdot R+V_{IN}$	$V_{OUT}=V_{IN}\cdot \left(1+\frac{100K\Omega }{R_{G}}\right)+V_{REF}$
$R_{GSR}=\frac{1}{C_{GSR}}$	$R_{GSR}=\;\frac{\frac{V_{IN}}{V_{CC}-V_{REF}}\cdot R3\cdot \left(R1+R2\right)+R3\cdot R2}{R1-\frac{V_{IN}}{V_{CC}-V_{REF}}\cdot \left(R1+R2\right)}$
$C_{GSR}=\;\frac{V_{OUT}-V_{IN}}{V_{IN}\cdot R}$	$C_{GSR}=\frac{1}{R_{GSR}}$

**Table 5:** Relevant results of the stationary analysis of the GSR signals.

	PeH	PhyAS
ADC range	[0, 5] V	
Signal offset	0.5 V	0.1786 V
Amplification limit	3.3 V	5 V
% ADC	55.96%	96.42%
ADC bits	10 bits (1,024 levels)	24 bits (16,777,216 levels)
Level of quantification	4,882,812.5 nV	29.8 nV
Measurable values	573	16,178,029
Measurable $R_{GSR}$ range	[≈17.86 KΩ, ≈14.61 MΩ]	[≈0.19 Ω, ≈13.5 MΩ]
Measurable $C_{GSR}$ range	[≈68.43 nS, ≈55.98 µS]	[≈74.07 nS, ≈5.19 S]

**Table 6:** Statistics of the resolution of GSR signals for both acquisition modules.

	∆R_GSR_				∆C_GSR_			
	Min	Max	Mean	STD	Min	Max	Mean	STD
PeH	31.24 Ω	8.59M Ω	25.51 KΩ	374.83 KΩ	97.75 nS	97.75 nS	97.75 nS	0
PhyAS	0.25 Ω	2.71 Ω	0.81 Ω	0.6 Ω	0.015 pS	2.96 S	2.47 µS	5.97 mS

**Table 7:** Amplitude of noise in PeH and levels of the ADC that it occupies.

	Amplitude	ADC Levels	% Measurable Range
Maximum	4.12 µS	42.14	7.37%
Mean	3.29 µS	33.64	5.88%
Minimum	1.98 µS	20.29	3.55%
STD	1.1 µS	22.52	3.94%

**Table 8:** Comparison of the different designs of the pulse sensor.

	PeH	PhyAS
Subjection	Clip on the finger	Clip on the ear lobe
Operating principle	PPG transilluminated	PPG reflection
Signal	Unknown (it is obtained from the sensor’s display)	Signal of the photoreceptor, which is processed, and the maximums are obtained to obtain the value of instantaneous pulse variability
BPM representation	Integer	Double (2 digits decimals)

**Table 9:** Comparison of the different designs of the temperature sensor.

	PeH	PhyAS
Sensor type	RTC	FIR
Skin contact	Yes	No (only proximity)
Signal conditioning	The stages are a Wheatstone bridge and an instrumentation amplifier.	Unknown (inside the sensor)
Acquisition	Arduino ADC	Arduino I2C (ADC is inside the sensor)

**Table 10:** Response time.

Signal	Signal Increase (°C)	Response Time (s)	Response Speed (°C/s)	Correlation
PeH	3.005	62.1	0.048	0.805
PhyAS	1.687	3.3	0.51	0.986

**Table 11:** Noise level.

Signal	Average Noise (°C)	STD Noise (°C)	Noise Range (°C)
PeH	0.39	0.3	[0.09, 0.69]
PhyAS	0.12	0.04	[0.08, 0.16]

**Table 12:** Comparison between PhyAS and PeH.

Sensor	Explanation
GSR	The PhyAS conditioning stage is best suited to the signal acquisition stage. This is because both stages work the signals in the same voltage range. For this reason, PeH wastes 34% of the ADC quantification levels. Both platforms add an offset to the signal that also wastes part of the converter, being the most affected PeH with 10% and PhyAS with 3.58%. This means that the percentage of ADC quantifier levels used are 96.42% for PhyAS while 55.96% for PeH. The number of measurable values is 28,234 times greater for PhyAS than for PeH, due to the 24 bits conversion of PhyAS versus 10 of PeH. The range measurable by PhyAS comprises 8 orders of magnitude while PeH comprises 3 orders of magnitude.
Pulse	No signal quality is lost with respect to PeH. PhyAS gets higher resolution. PhyAS can adapt the window size as needed, in search of a better emotional detection, having the possibility of using the instantaneous values.
Temperature	Both signals are similar to the pharmacy thermometer signal. The correlation of PhyAS is higher and almost the unit with a value of 0.986 against the correlation of PeH with a value of 0.805. The response time of the sensor is improved. While in PeH it is needed to wait a little more than a minute until the sensor value stabilizes at body temperature, using PhyAS, you only have to wait a little more than 3 s. It is noticeable the noise level present in PeH is higher than that of PhyAS.
